# Frequency- and State-Dependent Network Effects of Electrical Stimulation Targeting the Ventral Tegmental Area in Macaques

**DOI:** 10.1093/cercor/bhaa007

**Published:** 2020-04-09

**Authors:** Sjoerd R Murris, John T Arsenault, Wim Vanduffel

**Affiliations:** 1 Department of Neurosciences, Laboratory of Neuro- and Psychophysiology, KU Leuven Medical School, Leuven 3000, Belgium; 2 Leuven Brain Institute, KU Leuven, Leuven 3000, Belgium; 3 Athinoula A. Martinos Center for Biomedical Imaging, Massachusetts General Hospital, Charlestown, MA 02129, USA; 4 Department of Radiology, Harvard Medical School, Boston, MA 02144, USA

**Keywords:** Anesthesia, fMRI, *Macaca mulatta*, microstimulation, VTA

## Abstract

The ventral tegmental area (VTA) is a midbrain structure at the heart of the dopaminergic system underlying adaptive behavior. Endogenous firing rates of dopamine cells in the VTA vary from fast phasic bursts to slow tonic activity. Artificial perturbations of the VTA, through electrical or optogenetic stimulation methods, generate different and sometimes even contrasting behavioral outcomes depending on stimulation parameters such as frequency, amplitude, and pulse width. Here, we investigate the global functional effects of electrical stimulation frequency (10, 20, 50, and 100 Hz) of the VTA in rhesus monkeys. We stimulated 2 animals with chronic electrodes, either awake or anesthetized, while concurrently acquiring whole-brain functional magnetic resonance imaging (fMRI) signals. In the awake state, activity as a function of stimulation frequency followed an inverted U-shape in many cortical and subcortical structures, with highest activity observed at 20 and 50 Hz and lower activity at 10 and 100 Hz. Under anesthesia, the hemodynamic responses in connected brain areas were slightly positive at 10 Hz stimulation, but decreased linearly as a function of higher stimulation frequencies. A speculative explanation for the remarkable frequency dependence of stimulation-induced fMRI activity is that the VTA makes use of different frequency channels to communicate with different postsynaptic sites.

## Introduction

The ventral tegmental area (VTA) is a midbrain structure at the heart of the dopaminergic (DA) system which sends projections throughout most of the brain (Berger et al. 1988; Mark Williams and Goldman-Rakic 1998). The firing rate of DA VTA neurons ranges from low frequency, tonic activity (<10 Hz) to high frequency, phasic bursts (20–100 Hz). Phasic VTA modulations have been causally linked to cue-driven reward seeking, Pavlovian conditioning, and operant conditioning (Tsai et al. 2009; Adamantidis et al. 2011; Steinberg et al. 2013; Arsenault et al. 2014; Stauffer et al. 2016), while decreased tonic firing has been shown to reinstate extinction learning (Chang et al. 2015). Relatively little is known, however, about the relationship between firing frequency and activity modulation at projection sites. Presumably, this temporal component of the VTA responses is key to understanding the wide range and sometimes contradictory behavioral effects related to dopamine signaling (Schultz 2007).

One way to investigate how the temporal dynamics of a given brain region causally affects connected areas is by stimulating a region either electrically (Ekstrom et al. 2008) or optogenetically (Gerits et al. 2012), while measuring its functional consequences in target regions. For example, electrical microstimulation (EM) of the lateral geniculate nucleus (LGN) in macaques generated positive and negative functional magnetic resonance imaging (fMRI) signals in area V1 at high (100–200 Hz) and low frequencies (3–48 Hz), respectively (Logothetis et al. 2010). Surprisingly, modulations of opposite polarities were found when stimulating the ventral thalamus in pigs (Paek et al. 2015), with low (10 Hz) frequencies causing positive hemodynamic signals in M1, while high (130 Hz) frequencies generated negative modulations. Such inconsistent results indicate that the effect of EM on distal structures may depend on several factors including the frequency of stimulation, the region stimulated, and the downstream structure being monitored. Moreover, the diversity observed indicates that it is difficult to predict brain-wide effects of electrically stimulating any particular region and that these relationships must be tested empirically.

The effect of stimulation frequency on physiology and behavior has been most extensively studied in rodents. Tsai and colleagues demonstrated that low (1 Hz) versus high (50 Hz) frequency optogenetic stimulation-induced tonic and phasic burst-like patterns within dopamine neurons, respectively (Tsai et al. 2009). Moreover, they found that spatial locations paired with high frequency stimulation-induced place preferences, while low frequency stimulation did not. In contrast, self-administration of ethanol was found to decrease only with low-frequency stimulation mimicking tonic activity (5 Hz), but not at higher frequencies (50 Hz) (Bass et al. 2013). The differential effectiveness of stimulation frequencies in modulating distinct behaviors suggests that the functional ramifications of VTA stimulation on downstream structures are also frequency-dependent. Two studies have investigated the widespread effects of VTA stimulation in rodents using optogenetics and fMRI (ofMRI) (Decot et al. 2017; Lohani et al. 2017). These studies have demonstrated that neuronal DA activity is altered in a widespread pattern not necessarily confined to regions with the strongest anatomical connections, such as the ventral striatum. Both studies focused on a single stimulation frequency (30 Hz/10 s (Decot et al. 2017); 20 Hz/20 s (Lohani et al. 2017)), however, so possible frequency-dependent effects were not systematically explored. An additional constraint in most rodent ofMRI studies is that animals are anesthetized. Considering the marked changes in functional connectivity between the awake state and at different levels of anesthesia both in monkeys (Uhrig et al. 2018) and rodents (Paasonen et al. 2018), it is plausible that the state of the animal affects stimulation responses. Moreover, the capacity of VTA stimulation to trigger reanimation from either isoflurane- or propofol-induced anesthesia, underscores possible state-dependent effects of stimulation (Solt et al. 2014; Taylor et al. 2016). In addition, DA innervation differs greatly between rodents and primates, indicating that species differences likely also influence the effects of VTA stimulation (Berger et al. 1988).

Despite the clinical and experimental relevance of brain-stimulation parameters, we are not aware of any systematic investigation into the effects of EM frequency or the state of the animal on cortical and subcortical signal propagation (but see Logothetis et al. 2010). Previously, we have demonstrated that EM of the VTA (VTA-EM) in awake macaques leads to increased hemodynamic responses in brain areas commonly associated with reward (Arsenault et al. 2014). Stimulation parameters in that study were based on cortical stimulation in the monkey, employing a stimulation frequency of 200 Hz (Ekstrom et al. 2008). In the current study, the intent was to identify effects of VTA-EM at more biologically relevant frequencies (10–100 Hz) on brain-wide fMRI activation patterns in both awake and anesthetized monkeys.

## Materials and Methods

### Subjects

Two male rhesus macaques (*Macaca mulatta*) participated in the current study (Monkey T: ~ 8.5 kg, 7 years old; Monkey D: ~ 6.5 kg, 6 years old). The animals are socially housed in groups in enclosures exposed to daylight and are provided with toys and enrichment devices at the primate facility of the KU Leuven Medical School. Food is available *ad libitum* and monkeys can drink water until satiated during experiments and training. Both monkeys underwent initial surgery to implant an MR-compatible headset attached to the skull with ceramic screws that is subsequently covered with dental cement, as previously described in detail (Vanduffel et al. 2001). The 2 monkeys participated in other experiments in which we combined VTA-EM with behavioral paradigms, and awake contrast-agent-enhanced fMRI (Arsenault and Vanduffel 2019). Animal care and all surgical and experimental procedures have been approved by the ethical committee of the medical school at KU Leuven and conform to national and European guidelines (Directive 2010/63/EU).

### VTA Electrical Microstimulation

**Figure 1:**
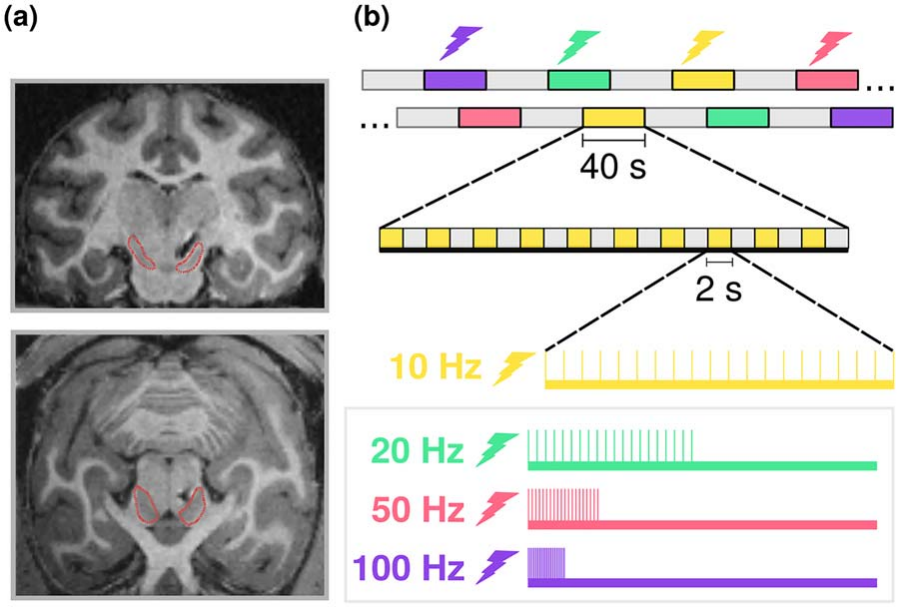
(*a*) Coronal and transverse slices of a post-operative T1-weighted anatomical scan of Monkey T. Black distortion indicates the position of the chronic electrode relative to the Substantia Nigra, indicated by the red outline. (*b*) Overview of the fMRI design consisting of alternating blocks of 40 seconds of stimulation (10 Hz, yellow; 20 Hz, green; 50 Hz, red; 100 Hz, purple) and baseline (no stimulation, gray). Each of the stimulation blocks consisted of 10 equally-spaced stimulation trains of the same frequency.

The details of the VTA-EM procedure have been published elsewhere (Arsenault et al. 2014). In brief, both monkeys were chronically implanted with micro-brush electrode arrays (Microprobes) that consist of 32 microwires (25–50 μm thickness) (McMahon et al. 2014). T1-weighted images were obtained on a 3 T horizontal bore full-body scanner (Siemens) for pre-operative planning, peri-operative guidance, and post-operative confirmation of electrode positioning within the VTA (Fig. 1*a*). For stimulation, an 8-channel digital stimulator (DS8000, World Precision Instruments) was connected to stimulus isolators (DLS100, World Precision Instruments) that were in turn connected to the micro-brush electrode array connector (Omnetics) attached to the monkey’s headpost. The current isolators were used to ensure that the amplitude of current delivered to the VTA electrodes was consistent across electrodes and time. The timing of the electrical pulse trains was generated using custom Python scripts (Python) and the stimulus isolator was used in current instead of voltage mode. Biphasic pulses were triggered consisting of an anodal (positive) leading pulse of 0.2 ms followed by a cathodal (negative) 0.2 ms pulse (0.4 ms pulse width). We chose anodal leading pulses based on previous studies in which we successfully employed EM in the macaque (Ekstrom et al. 2008; Arsenault and Vanduffel 2019). The effect of the AC frequency of the stimulation pulses within a train was the main thrust of our current investigation, which we will address in further detail below.

### Animal Behavior

In preparation for the concurrent VTA-EM fMRI experiments, monkeys were trained with operant conditioning techniques. Subjects were required to fixate upon a central white dot on a gray background in a mock setup resembling the MR-scanner environment. This mock setup included recorded scanner noise and a cylinder similar to the magnet’s bore. Fixation was monitored at 120 Hz using an infrared-based eye-tracking system (ISCAN). In the scanner, the same fixation spot as used during training was projected onto a translucent screen placed 57 cm from the monkey’s eyes, using a Barco LCD Projector (Barco).

### Online Fixation Performance

During training and awake scanning sessions, online fixation performance was monitored and monkeys were rewarded with small drops of juice for maintaining fixation on the white dot within a virtual fixation box (Supplementary Material S1). The mean fixation performance was calculated over runs and used to reject fMRI runs where the point of gaze remained in the fixation box less than 90% of the time.

### Post hoc Eye-Position Analysis

Horizontal and vertical visual coordinates of eye movements from the ISCAN eye-tracking system were analyzed with custom-made scripts in MATLAB (Mathworks). Eye blinks were first removed from the horizontal and vertical traces to ensure that eye tracking data reflected actual eye position. For each stimulation frequency, the mean eye position during each run was then calculated for all time points within a time window, 200 ms before to 2000 ms after stimulation onset. We then compared eye positions across different frequencies at each time point using a one-way ANOVA, including a Bonferroni correction for multiple comparisons across time points.

### fMRI Parameters

fMRI data were acquired using echo-planar imaging (EPI) sequences with a voxel size of 1.25 × 1.25 × 1.2 mm. We collected volumes of 50 slices with an interleaved slice acquisition. The repetition time (TR) for each acquisition was 2000 ms with an echo time (TE) of 13 ms. We used an external transmitter- and 8 channel receiver-coils placed just above and lateral to the monkey’s head, respectively. A gradient echo scan was acquired each session to validate online that all coils were functional (voxel size: 1.25 × 1.25 x 1.2 mm/TR: 5 ms/TE: 2.03 ms). Before each scanning session, we manually shimmed the magnetic field.

### EM-fMRI Experiments

For both monkeys, we ran 7 VTA-EM fMRI sessions: 5 during which the subjects were awake and fixating, and 2 under ketamine anesthesia. To measure contrast-enhanced changes in cerebral blood volume (CBV), we injected the monkey’s femoral/saphenous vein with an iron-based contrast agent (9–10 mg/kg, Molday ION, BioPAL). We subsequently fixed the monkey’s head to an MR-compatible plastic chair in a sphynx position (Vanduffel et al. 2001; Leite et al. 2002). The VTA electrode array connector embedded in the headpost was attached to the isolators via the filter panel of the scanner room. The digital stimulator and isolators where located outside the scanner room to reduce radiofrequency noise. In addition, the connector and the wires leading to the connector were shielded with copper tape. After placing the transmitter- and receiver coils close to the monkey’s head, we moved the animal into the bore, positioning the head in the isocenter of the magnet. Thereafter, we measured the impedance of 2 selected channels in each monkey used for EM with an LCR Meter (B&K Precision, Yorba Linda). The impedance for the 2 channels used in monkey T ranged from 50 to 300 kΩ measured at 100 Hz, with monopolar stimulation of 2 electrodes and utilizing a ground wire positioned between the skull and dura mater. Monkey D did not have an implanted ground wire because of technical issues, so we measured the impedance across the 2 channels, obtaining values between 50 and 200 kΩ. This also meant that microstimulation in monkey D was bipolar across the 2 selected electrodes. Prior to the awake sessions, the eye camera signal was calibrated to convert the digital eye positions into visual degrees. Because of the close proximity of the VTA to the oculomotor nerve and nuclei, saccadic eye movements could be used as an indication of stimulation. Before each scan we tested whether the microstimulation set-up was operational and confirmed the functional impact of VTA-EM by slowly increasing the current to determine a saccade threshold. VTA-EM current levels were then kept below the saccade threshold to avoid current spread to sites outside the VTA and to avoid eye movements.

### Awake VTA-EM fMRI

In the awake experiment (5 sessions, 65 runs in monkey T; 5 sessions, 103 runs in monkey D), we opted for a block design to increase detection power (Liu et al. 2001). This was selected after a pilot study, using an event-related design and performed with the same animals, in which we failed to elicit major stimulation evoked activity, even at higher stimulation frequencies (100 Hz). Each run of the block design consisted of 355 TRs (TR = 2 s, 12 min total duration). The monkeys continuously performed the previously described passive fixation task during each run (see Animal Behavior). After an initial baseline period of 20 TRs, stimulation blocks of 20 TRs (40 s) alternated with non-stimulation blocks of equal length (Fig. 1*b* provides a visual overview of the design). The timing of the stimulation pulses did not correlate with the juice rewards, to exclude the influence of motivational and reward signals on the observed activity patterns. There were 4 different types of stimulation blocks (10, 20, 50, and 100 Hz) that each occurred twice during a run. The order of the different stimulation-frequency blocks was pseudo-randomized to counter possible summation and anticipation effects. Within a given stimulation block, pulse trains were triggered at the onset of every odd TR. In these experiments, we aimed to examine the effect of stimulation frequency on fMRI activity, while controlling for the amount of current delivered. We therefore normalized the number of pulses in each block by inversely relating stimulation frequency to stimulation duration (10 Hz, 2000 ms; 20 Hz, 1000 ms; 50 Hz, 400 ms; 100 Hz, 200 ms). The amplitude for VTA-EM was set at half the saccade threshold, which was determined individually before the onset of each session (ranging 190–210 μA in monkey T; 70–170 μA in monkey D).

### Anesthetized VTA-EM fMRI

In the second experiment, we repeated VTA-EM fMRI using the same electrical stimulation paradigm as in the awake block design above (2 sessions, 32 runs in monkey T; 2 sessions, 36 runs in monkey D). The monkeys were initially anesthetized by intramuscular administration of a mixture of 25 mg Ketalar (ketamine hydrocholoride) and 0.5 mg Domitor (medotomidine hydrochloride). The same mixture was administered every 45 min intravenously through a catheter placed in the femoral vein. The monkeys were video monitored during experiments and a heating pad was placed on their side to minimize fluctuations in temperature. We further assessed the depth of anesthesia between runs by manually checking muscle tension and the monkey’s breathing patterns and readjusted the amount of anesthetic to maintain the monkey in a stable state. We were not able to detect any eye movement induced by stimulation at amplitudes up to 1000 μA in the anesthetized state. Given the absence of this behavioral signal, we decided to set the stimulation amplitudes to approximately twice the average saccade threshold of those used in the awake experiments (744 μA in monkey T; 488 μA in monkey D) or ~ 4 times higher currents than in the awake experiments. Note that in a previous EM-fMRI study targeting parietal cortex (Premereur et al. 2015) highly similar fMRI activations were found during awake and anesthetized scans when anesthetized currents were 5 times those of the awake currents.

### fMRI: Pre-Processing & General Linear Model

The following data acquisition pre-processing steps were performed using custom MATLAB scripts and SPM 12 (Statistical Parametric Mapping, University College London). Runs in which the monkey fixated less than 90% of the time within the specified fixation window, based on online fixation measurements (Supplementary Material S1), were discarded from further analysis, as were runs containing apparent ghosting in the EPI’s. For each remaining run, we applied a 3D slice-by-slice motion correction algorithm (Kolster et al. 2009). Secondly, we corrected for motion between runs by realigning them to a template derived from the same session. We subsequently performed linear and non-linear co-registration of each functional run, warping them to an anatomical template of the individual monkey (JIP fMRI Analysis Toolkit, J. Mandeville; www.nitrc.org/projects/jip). Following co-registration to the individual monkey’s anatomy, we registered functional data from both animals to the D99 template macaque brain (D99-space: Reveley et al. 2017). Finally, we spatially smoothed the functional data in SPM employing an isotropic Gaussian kernel (FWHM: 1.5 mm). The runs were analyzed by fitting a general linear model (GLM: Worsley and Friston 1995). Timing of the VTA-EM stimulation blocks were used as regressors of interest together with the non-stimulated blocks as a baseline condition. These regressors were fitted as boxcar models convolved with an iron oxide contrast agent response function (Vanduffel et al. 2001). When using an iron-based contrast agent, increased CBV results in decreased MR signal intensity (Leite et al. 2002). To account for this, we inverted the polarity of contrasts when computing contrast matrices. Accordingly, positive *t*-scores indicate regions with elevated CBV. We compiled runs from the 2 monkeys in a fixed-effects group analysis in which we equalized the contributions of each monkey by using an equal number of runs (awake: 5 sessions, 60 runs in each monkey; anesthetized: 2 sessions, 30 runs in each monkey). This analysis was performed in D99 template space^22^. To assess the consistency of the activity patterns across monkeys, we executed a conjunction analysis (Friston et al. 2005). The conjunction analysis ($P<0.005\Big)$ thresholds significant activations as those that survive a predefined statistical threshold ($P<\sqrt{0.005}$) in both of the animals.

### fMRI: ROI Analysis

Further analysis of the group data was performed using 181 cortical and subcortical anatomical regions of interest (ROIs) available for each hemisphere in the D99 space. ROIs were based on the Saleem and Logothetis parcellation scheme in D99 space, since these include many subcortical regions. The D99 scheme lacked ROIs for the VTA and the nucleus accumbens (NAc: a subregion of the striatum with strong DA innervation). We therefore added these anatomical ROIs manually, increasing the number of ROIs per hemisphere to 183. It is noteworthy that the visual ROIs deviate from those based on previous retinotopic mapping experiments of our own laboratory (Janssens et al. 2014; Kolster et al. 2014; Zhu and Vanduffel 2019). The results of the present experiment, however, do not depend on the exact parcellation scheme used. We calculated mean *t*-score values across all voxels within a ROI for each run and for each of the 4 stimulation frequencies (10, 20, 50, and 100 Hz) relative to baseline. This analysis was performed on group data separately for the awake and anesthetized paradigms. ROI selection was then performed. ROIs used in further analysis had to fulfill one of 2 criteria: (1) any of the stimulation frequencies in either the anesthetized or awake paradigm resulted in a mean *t*-score higher than 2.3 (uncorrected at *P* < 0.01) across all voxels of the ROI or (2) at least 40 voxels in the ROI had *t*-scores higher than 2.3 (uncorrected at *P* < 0.01). The second requirement was added to include larger regions, in which focal VTA modulations were potentially reduced in strength after averaging across the region. To determine the subset of ROIs that were most affected by VTA-EM, we also used a stringent threshold of *t* > 3.7 (*P* < 0.0001) on the mean ROI *t*-scores.

Non-parametric statistical tests were used to compare *t*-score values from different conditions (Friedman’s Test & Wilcoxon Signed Rank Test). To get a better estimate of the relationship between frequency and fMRI activity across ROIs during the awake and the anesthetized runs, we compared a linear regression model of the effect of frequency (*T*-score ~ frequency) to a model with a nonlinear frequency factor added (*T*-score ~ frequency + frequency^2^) using the lmBF function from the BayesFactor R package (Rouder and Morey 2012). Bayes factors comparing the individual models to an intercept-only model were examined, as well as a comparison of the models with and without the nonlinear factor. Model selection was performed using these 3 models (intercept, linear, and nonlinear). In the absence of convincing evidence for the more complex model (Bayes factor > = 3), the simpler model can be selected as the most parsimonious explanation of the data (Jeffreys 1961).

To examine which ROIs exhibited similar activity patterns, we clustered ROIs based on their functional responses. We utilized functional data from the 4 different frequencies from the awake and anesthetized experiments (*t*-scores) for each of the ROIs. This functional data served as an input into an unsupervised k-means clustering algorithm, which groups ROIs based on a pre-defined number of clusters. We based the number of clusters on the data-driven Caliñksi–Harabasz criterion (Caliñski and Harabasz 1974).

### fMRI: Functional Connectivity

To investigate how functional connectivity changed between the awake and anesthetized fMRI experiments, we ran a seed-based functional connectivity analysis (Mantini et al. 2011) for each of the previously selected ROIs. Runs from the awake (Monkey T: *n* = 60, Monkey D: *n* = 60) and anesthetized (Monkey T: *n* = 30, Monkey D: *n* = 30) experiments were used to calculate and contrast separate functional connectivity matrices. We performed 2 pre-processing steps in addition to those described in our fMRI data analysis stream above (see fMRI: pre-processing & GLM section): (1) the application of a bandpass filter on each of the runs between 0.009 and 0.05 Hz and (2) regression of the cerebral spinal fluid (CSF) signal and its first derivative. An average time course for each of the ROIs was then obtained by averaging the signal across voxels. A subsequent functional connectivity map was calculated for each run by computing the Pearson correlation between all possible pairs of ROI time courses. Afterwards the run-based connectivity maps were transformed to Z-scores and combined in a fixed-effect analysis to establish group-level correlations. The resulting group Z-score maps were then reconverted into corresponding group correlation maps.

## Results

To investigate how stimulation frequency affects the pattern of elicited brain activity, we electrically stimulated the VTA via chronic electrodes (see Fig. 1*a*) using different frequencies (10, 20, 50, and 100 Hz), while measuring the corresponding neural responses in a block-design fMRI experiment. During the fMRI experiments, VTA stimulation blocks lasted for 40 s with brief stimulation trains within a stimulation block starting every 4 s (see Fig. 1*b*). To equalize the current delivered at each stimulation frequency, stimulation train duration was dependent on stimulation frequency with lower frequency trains lasting for longer time periods (see Materials and Methods).

### Fixation Performance

**Figure 2:**
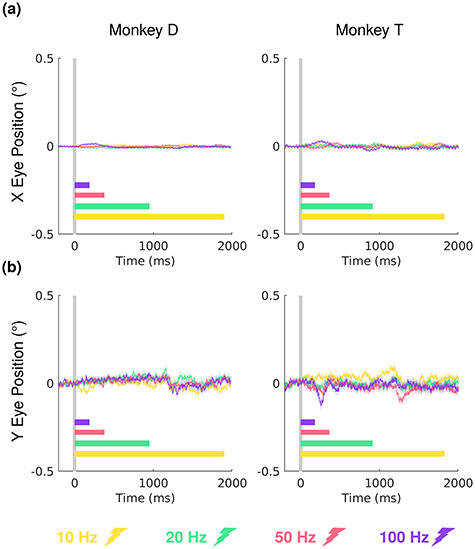
Eye positions of the 2 monkeys in all awake fMRI sessions: monkey D (5 sessions, 93 runs), monkey T (5 sessions, 65 runs). Mean (solid) and SEM (shaded) of horizontal (*a*) and vertical (*b*) eye position for each stimulation frequency 250 ms before stimulation onset (gray vertical line) to 2000 ms after.

Animals performed a passive fixation task during awake fMRI scanning sessions. Online measurements of fixation performance were used to control the animal’s behavior during scanning sessions and used to discard fMRI runs with fixation performance levels below 90%. After scanning was completed, a detailed post hoc analysis of eye position (horizontal and vertical eye traces) during VTA stimulation trains was performed to ensure that differences in eye movements could not account for the observed results (Fig. 2*a*,*b*). In addition, because of VTA’s proximity to the oculomotor nerve and the oculomotor nuclei, any EM that was not confined to the VTA would likely generate eye movements. In fact, small eye movements (+/− 0.5 visual degrees) were monitored in a pilot experiment using higher current levels in the same monkeys, indicating current spread outside the VTA (Supplementary Material S2). Most importantly, with the lower current levels used in this study (190–210 μA in monkey T; 70–170 μA in monkey D), no significant differences in eye position were found between frequencies in the time-window (−250 to +2000 ms) surrounding stimulation trains (one-way ANOVA, Bonferroni corrected, *P* > 0.05, see Fig. 2).

### Awake VTA-EM fMRI Activity Patterns

**Figure 3:**
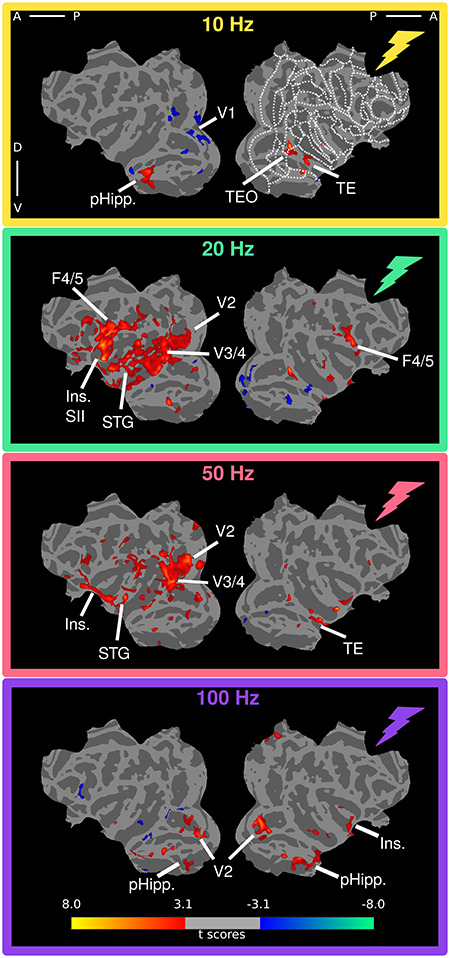
fMRI responses to VTA stimulation across frequencies in awake monkeys. *T*-score maps of the stimulation frequency versus baseline (uncorrected level, *P* < 0.001; cluster correction, 20) overlaid onto cortical flatmaps in the D99 template space (see methods). Regions showing significant activation are indicated on the maps. For a complete overview of ROIs and ROI abbreviations in D99 space see Supplementary Material S5.

First, we examined the effect of the frequency of VTA-EM on its brain-wide fMRI responses. More specifically, we examined whether changes in the frequency altered only the degree of activity modulation, or whether also, or even instead, if the regions that were affected might differ. Therefore, we first plotted VTA-EM-induced fMRI activity as function of frequency onto flattened cortical representations (uncorrected *P* < 0.001; cluster threshold 20, Fig. 3).

At 10 Hz, we found increased activity in inferotemporal (IT) and parahippocampal areas but also some reduced activity, primarily in early visual cortical regions. About 20 Hz stimulation resulted in more extensive increases in activity, covering many more occipito-temporal, premotor, and prefrontal areas of the hemisphere contralateral to the stimulating electrodes. In general, the stronger effects observed in the contralateral hemisphere may be explained by reduced temporal SNR ipsilaterally due to the presence of the recording well which results in greater distances between the coil and brain tissue on that side (Supplementary Material S3). Modulation of activity was especially pronounced in contralateral visual cortical regions (including areas V2, V3, and V4), areas along the upper and lower bank of the entire extent of the STS, areas in STG, insular and gustatory cortex, as well as motor, pre-motor, and orbito-frontal areas (12, 13). In the ipsilateral hemisphere, increased activity was mostly confined to motor/premotor areas (F1, F4/5) and IT, but deactivations were also observed in other portions of IT and parahippocampal cortex. Stimulation at 50 Hz resulted in attenuated activations compared with 20 Hz, although there is a high degree of overlap for activations in earlier visual regions of the contralateral hemisphere (V2, V3, and V4). In addition, activation was relatively strong in the anterior insula and secondary gustatory regions. The extent of cortical surface affected by VTA-EM relative to the 20 Hz stimulation became even smaller at 100 Hz. There was also a substantial shift of significantly activated regions to more ventral sites, including parahippocampal areas, the STG, and insular cortex. A conjunction analysis shows that the general fMRI activity patterns are largely consistent across animals and are not driven by exceedingly strong modulations in one of the 2 monkeys (Supplementary Material S4a). The symmetrical activation of the parahippocampal cortex for the 10 and 100 Hz conditions and in particular an overall increase in activity in most of the contralateral hemisphere at 20 Hz, and to a lesser extent at 50 Hz, were corroborated by the conjunction analysis.

### Anesthetized VTA-EM fMRI Activity Patterns

Since the majority of electrical stimulation studies with concurrent fMRI are performed in anesthetized animals, we also investigated the state-dependent effects of VTA stimulation by comparing induced fMRI activity in anesthetized versus awake preparations. We anesthetized monkeys with the NMDA antagonist ketamine (see Materials and Methods) and applied stimulation protocols identical to those of the awake experiments, except that higher currents were used. The current was increased based on an earlier cortical stimulation study, using a similar anesthesia protocol, which demonstrated that higher currents were needed under anesthesia to generate activations roughly equivalent to those of awake preparations (Premereur et al. 2015). Given these data and those found in an earlier study that stimulated cortical and subcortical regions in awake and anesthetized monkeys (Logothetis et al. 2010), we hypothesized that, under anesthesia, VTA stimulation should elicit a pattern of activation similar to that found in the awake state.

**Figure 4:**
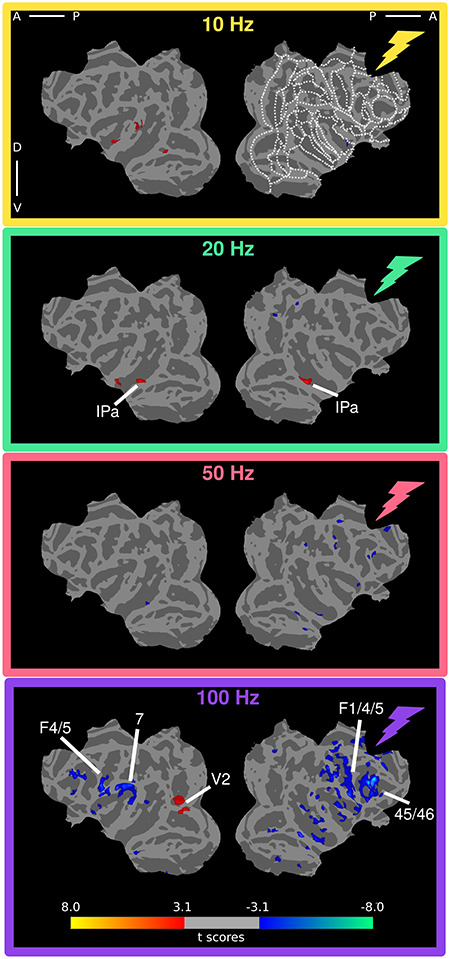
fMRI response to VTA stimulation across frequencies in anesthetized monkeys. *T*-score maps of the stimulation frequency versus baseline (uncorrected level, *P* < 0.001; cluster correction, 20) overlaid onto cortical flatmaps in the D99 template space (see Materials and Methods). Regions showing significant activation are indicated on the maps. For a complete overview of ROIs and ROI abbreviations in D99 space see Supplementary Material S5.

Contrary to our prediction, however, VTA stimulation under ketamine anesthesia induced considerably different patterns of cortical modulation compared with the awake state (Fig. 4). At 10 Hz stimulation, significant cortical modulations were primarily restricted to cortex contralateral to the stimulating electrode in posterior IT, the dorsal bank of the STS and STG. At 20 Hz, modulations were again largely positive with activations in IT. At 50 Hz, no significant activations were observed on the cortical surface. In contrast, patchy deactivations were distributed across the cortical surface ipsilateral to the stimulating electrode in the lower bank of the STS, somatosensory cortex, IPS, F1, and areas 24 and 46. Finally, 100 Hz stimulation induced multiple strong deactivations across the cortical surface, with the strongest and most extensive deactivations found ipsilateral to the electrode. These negative modulations were most pronounced in frontal regions (8, 44, 45, and 46) and somatosensory and premotor cortical areas. Overall, fMRI modulations induced by VTA stimulation grew increasingly negative with increasing stimulation frequencies in anesthetized animals. A further conjunction analysis of the anesthetized data indicated that the patterns of modulation across frequencies were largely consistent across the 2 animals (Supplementary Material S4b). Deactivations were mostly absent in the 10 and 20 Hz conditions and were increasingly stronger in the 50 and especially in the 100 Hz condition. This relationship does not match the inverted U-shaped curve observed for awake animals, suggesting that anesthesia substantially alters the effects of subcortical stimulation on fMRI responses. It is noteworthy that the tSNR for the anesthetized dataset and the strength of the fMRI modulations were more symmetric across hemispheres (Supplementary Material S3).

### ROI Analysis

To gain a more quantitative insight into the amplitude and spatial extent of VTA-EM-induced activity modulation, we performed a detailed ROI analysis of all stimulation frequencies for both the awake and anesthetized preparations. To select the most relevant ROIs from the 181 anatomical ROIs in each hemisphere of the D99 atlas (Reveley et al. 2017), we chose ROIs for further analysis that displayed significant modulations at any of the stimulation frequencies (uncorrected *P* < 0.05, see Materials and Methods). A total of 101 ROIs fulfilled this criterion (for an overview of these ROIs see Supplementary Material S5). In addition, we included anatomical VTA and NAc ROIs, because of their importance in DA signaling. These 2 ROIs did not survive the chosen thresholds however. The lack of significant subcortical modulations may result from poorer tSNR in subcortical regions, as these regions are farther from the MRI receive coils and may experience signal loss due to their proximity stimulating electrode (Supplementary Material S3).

**Figure 5:**
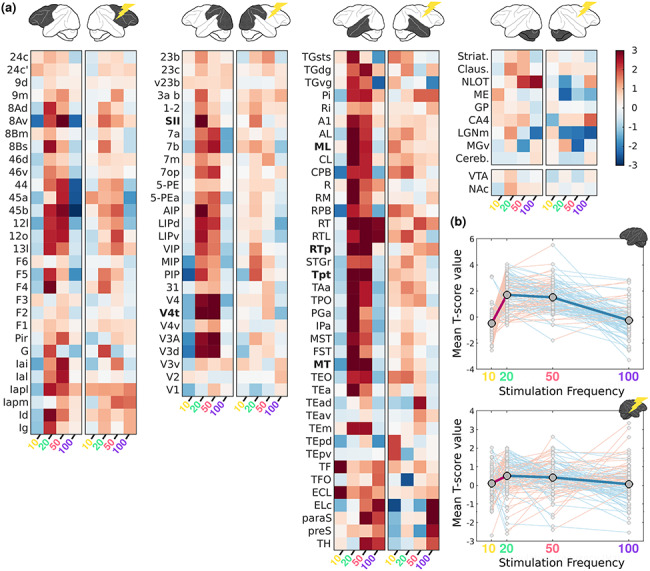
ROI analysis of VTA stimulation-induced activity as function of frequency in awake monkeys. (*a*) Heat maps of fMRI activity (mean *t*-score values) for ROIs in the left and right hemispheres as function of stimulation frequency. ROIs are derived from the D99 atlas and divided into: frontal, parietal-occipital, temporal and subcortical subdivisions (top left to right). The small yellow lightning icon marks the stimulated hemisphere. ROI names in bold are those in which the strength of the activity survived a stringent statistical threshold (*P* < 0.0001) for one of the frequencies and can be considered most affected by VTA-EM (*b*) Mean *t*-score values (gray circles) for anatomical ROIs combined for the left, unstimulated, (top) and right, stimulated, hemispheres (bottom). Connecting lines between each of the ROIs indicate an increase (pink) or decrease (light blue) in activity between neighboring stimulation frequencies. The thick central line and larger circles show the mean values across all ROIs.

The relationship between stimulation frequency and fMRI response measured in awake fixating monkeys is plotted for each ROI in Figure 5*a*. The average relationship between stimulation frequency and the strength of fMRI activity can best be described as an inverted U-shape, with patterns of VTA-EM-induced activity for 10 and 100 Hz being more similar than those obtained at 20 and 50 Hz. Regions most strongly affected by VTA-EM (*P* < 0.0001: see Methods) include higher visual areas spanning extrastriate visual areas (V4t, MT), parietal cortex (Tpt), somatosensory cortex (SII), and secondary auditory regions (RTp, ML). In the majority of the ROIs, stimulation at 20 and 50 Hz led to higher activation levels than did the 10 and 100 Hz stimulation (Fig. 5*b*). In both hemispheres, a main effect of *stimulation frequency* was found (Friedman’s Test; Right: *P* = 10^−6^ and Left: *P* = 10^−33^). A post hoc Wilcoxon Signed Rank Test revealed that the most substantial differences were observed between 10 and 20 Hz (Right: *P* = 10^−6^ and Left: *P* = 10^−16^), 10–50 Hz (Right: *P* = 0.0023 and Left: *P* = 10^−16^), 20–100 Hz (Right: *P* = 10^−4^ and Left: *P* = 10^−14^) and 50–100 Hz (Right: *P* = 10^−4^ and Left: *P* = 10^−16^). In contrast, there was no significant difference between 10–100 Hz (Right: *P* = 0.060 and Left: *P* = 0.054) and 20–50 Hz (Right: *P* = 0.62 and Left: *P* = 0.39). To quantify the relationship between stimulation frequency and fMRI activity, we fit linear regression models with either a purely linear relationship (*t*-score ~ frequency) or one with the addition of a nonlinear factor (*t*-score ~ frequency + frequency^2^). Bayes factor (BF) was then used to determine the most parsimonious model. We first compared each model with an intercept-only model. This yielded good evidence for the nonlinear model (BF = 752.21). In contrast, the evidence suggested that the intercept-only model described the data better than the linear model (BF = 0.11). Accordingly, the nonlinear model was also strongly favored over the linear model (BF = 6661.63) when they were directly compared. These results confirm that a nonlinear relationship between stimulation frequency and brain-wide fMRI responses best describes the observed data.

**Figure 6:**
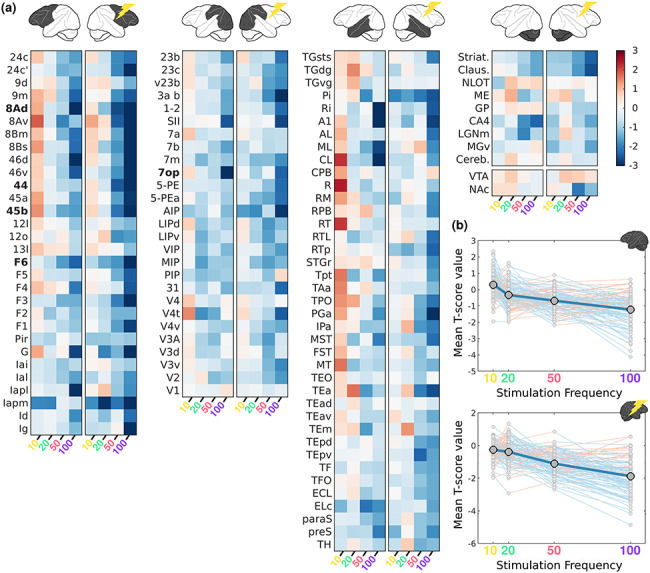
ROI analysis of VTA stimulation-induced activity as function of frequency in sedated monkeys. (*a*) Heat maps of activity (mean *t*-score values) for ROIs in the left and right hemispheres as function of stimulation frequency. (*b*) Mean *t*-score values (gray circles) for anatomical ROIs combined for the left, unstimulated, (top) and right, stimulated, hemispheres (bottom). Connecting lines between each of the ROIs point to an increase (pink) or decrease (light blue) in activity between neighboring stimulation frequencies. Conventions as in Figure 5.

In anesthetized monkeys, the ROI analyses revealed a substantially different pattern of modulation as function of stimulation frequency (Fig. 6*a*). At lower frequencies (10–20 Hz), the mean *t*-values fluctuate just above zero, while at higher frequencies (50–100 Hz) strong deactivations emerged in ROIs of frontal (areas 46, 8, 44, 45, F3, G, and Insular areas), parietal (SII, area 1–2, 3ab, 23, and AIP), and temporal cortex (TEa, IPa, and PGa), in addition to some subcortical regions (Striatum and Claustrum). Thus, the average fMRI response across ROIs displays a negative relationship with stimulation frequency, whereby increasing frequencies generate stronger deactivations (Fig. 6*b*). Strongest modulations (*P* < 0.0001; see Methods) are present in the parietal operculum (7op) and several frontal regions (44, 45b, 8Ad, and F6). As in the awake dataset, frequency exhibits a significant effect on fMRI activity in both hemispheres during the anesthetized condition (see Materials and Methods, Friedman’s Test; Right: *P* = 10^−34^ and Left: *P* = 10^−30^). A comparison of all possible pairs of stimulation frequencies showed significant differences for all pairs in both hemispheres (Wilcoxon Signed Rank Test, with *P*-values for all 12 combinations ranging between *P* = 0.028 and *P* = 10^−18^). As in the awake dataset, we assessed whether the fMRI response was better described by a linear (*t*-score ~ frequency) or nonlinear (*t*-score ~ frequency + frequency^2^) relationship. Comparison with an intercept-only model yielded strong evidence for both the linear (BF = 2.50 × 10^54^) and nonlinear models (BF = 1.33 × 10^54^), while comparisons between these models revealed anecdotal evidence for the linear over the nonlinear model (BF = 1.87). Because clear evidence is needed to justify a more complex model and thus avoid overfitting (i.e., addition of a nonlinear factor), this analysis indicates that the linear model provides the most parsimonious account of the anesthetized fMRI data. The divergence of the fMRI patterns in anesthetized and awake preparations, as suggested by the models above, is further supported by a formal comparison for each stimulation frequency when the monkeys are either awake or anesthetized. A relatively small (but significant) difference was found for the 10 Hz condition, in which fMRI activity was higher during anesthetized scans (Wilcoxon Signed Rank Test: *P* = 0.03). For the other 3 conditions (20, 50, and 100 Hz) the effect of *state* in comparisons of ROI distributions is very significant, with awake data showing much higher fMRI activity (Wilcoxon Signed Rank Test: *P* < 10^−25^, as can also be appreciated when comparing Figs 5*b* and 6*b*).

**Figure 7:**
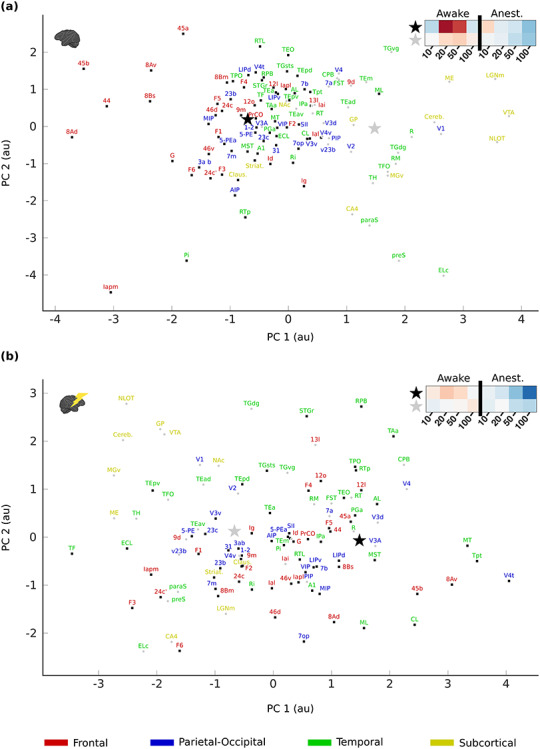
Clustering of the functional responses to VTA-EM. An unsupervised k-means clustering analysis on the ROI data of both the contralateral (*a*) and ipsilateral (*b*) hemispheres revealed 2 clusters in both cases. Individual ROIs from the 2 clusters are denoted by black (cluster 1) and gray (cluster 2) dots. Centroids of these clusters are represented by the stars in black (cluster 1) and gray (cluster 2). The functional profiles associated with the centroids are displayed in the top right of the 2 panels. The names of the ROIs are colored according to the large anatomical subdivisions of the brain they belong to (for a full overview of the ROIs: Supplementary Material S5). PC = Principal Component.

In the interest of revealing functional networks, we performed k-means clustering on the functional responses to the 4 different frequencies during both the awake and anesthetized experiments (*t*-scores) using the ROIs from the above analysis. We performed this analysis separately for each hemisphere. This allowed clusters to be compared between hemispheres, while controlling for the inter-hemispheric differences in the strength of responses. In both hemispheres, the optimal number of clusters, using the Caliñksi–Harabasz criterion, was 2. The first centroid of the 2 functional clusters in both the contralateral (Fig. 7*a*) and ipsilateral hemisphere (Fig. 7*b*) exhibits an inverted u-shape for the awake data and a linear decreased response with higher frequencies for the anesthetized data. The second functional cluster was associated with a similar but weaker decreased response as a function of frequency for the anesthetized data in both hemispheres. For the second cluster of the awake data, there was more diversity, with the contralateral hemisphere exhibiting a weaker u-shape curve, while the ipsilateral hemisphere showing a weak increase in activity as a function of frequency. Interestingly, the majority of frontal ROIs, many of which receive strong dopamine innervation (Berger et al. 1988), were grouped within the first cluster both in the contralateral (90%) and in the ipsilateral (87%) hemisphere. This suggests that in general, frontal regions are more strongly affected by VTA microstimulation. On the other hand, most subcortical ROIs are located in the second cluster (82% in both hemispheres). The distribution of ROIs into 2 clusters is less evident for the parietal-occipital (69% belong to cluster 1 in both hemispheres) and temporal cortex (left: 64% cluster 1; right: 68% cluster 1).

### Functional Connectivity: Ketamine-Dependent Effects

**Figure 8:**
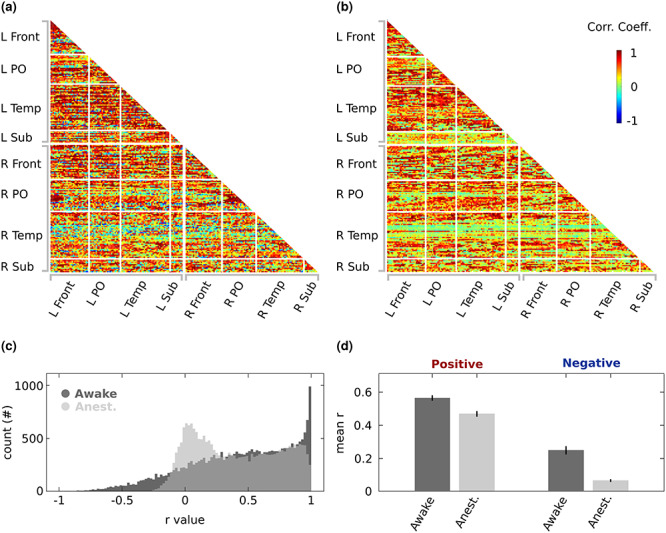
Functional connectivity profiles for the awake and anesthetized experiments. Temporal correlations between the fMRI signal in the ROIs listed in Supplementary Material S5, grouped in 4 broad anatomical subdivisions of the brain. Functional connectivity profile for the (*a*) awake (Monkey T: *n* = 60 runs, Monkey D: *n* = 60 runs) and (*b*) anesthetized (Monkey T: *n* = 30 runs, Monkey D: *n* = 30 runs) fMRI runs. The strength of the connections between ROIs are represented by color-coded correlation coefficients (r-values). (*c*) Comparison of the distribution of *r*-values for the awake and anesthetized scans. (*d*) Mean *r*-values for the positive and negative correlations. Error bars denote the bootstrapped 95% confidence intervals. L = left, R = right, Front = Frontal Cortex, PO = Parietal-Occipital Cortex, Temp = Temporal Cortex, Sub = Subcortical.

Studies in both rodents and monkeys comparing functional connectivity between awake and anesthetized animals have shown that several anesthetics affect the strength of correlated activity between brain regions (Barttfeld et al. 2015; Paasonen et al. 2018; Uhrig et al. 2018). Resting-state functional connectivity outcomes suggest that anesthesia decreases the flexibility of the network, through temporal dynamics that align more rigidly to the animal’s anatomical connections and by reducing negative correlations between regions. To investigate possible state-dependent effects, we generated functional connectivity maps for the awake (Fig. 8*a*) and anesthetized (Fig. 8*b*) sessions. In both the awake and anesthetized datasets, we found that the majority of regions displayed significantly correlated activity between regions belonging to both the same or different hemispheres (awake—84% of the ROIs and anesthetized—83%, FDR corrected *P* < 0.05). In line with previous studies (e.g., Uhrig et al. 2018), the distribution of interregional correlations in anesthetized scans showed less pronounced positive and negative correlations (Fig. 8*c*). To compare the strength of correlated activated between the awake and anesthetized data, we separated positive and negative correlations and compared the strength of correlations. Positive (permutation test, *P* < 10^−4^) and negative (permutation test, *P* < 10^−4^) correlated activity was stronger for the awake sessions (Fig. 8*d*).

## Discussion

Our results in primates demonstrate that both the frequency of electrical VTA stimulation and the state of the subject are important factors determining the pattern, amplitude, and even sign of brain-wide evoked hemodynamic activity. Frequency-dependent stimulation effects on fMRI activity as observed here have previously been established for other subcortical structures. However, those stimulation studies encompassed a broad range of brain structures, stimulation methods, animal models, and frequencies. These methodological differences complicate attempts to generalize the effects. The independent demonstration of changes in fMRI activity (whether in amplitude or polarity) related to changes in stimulation frequency within each of these studies does, however, emphasize the complexity of their relationship. It is important to note that the exact population of cells targeted by stimulation and their connectivity patterns affects the pattern of fMRI activity induced. Thus, caution is needed when comparing stimulation-induced activations across studies. This, however, also emphasizes the benefits of restricting the parameter space and examining the effect of a single feature of stimulation (such as frequency in the present study) on neural responses. The complex contribution of stimulation frequency to effective connectivity is exemplified by contrasting 2 electrical stimulation studies targeting thalamic structures in larger mammals. After high-frequency stimulation (100 Hz) of primate dLGN, the authors of this study observed positive and negative BOLD responses in area V1 and extrastriate visual cortex, respectively (Logothetis et al. 2010). Low-frequency stimulation (12 Hz) yielded highly similar results in extrastriate cortex, but a negative BOLD signal in area V1. In contrast, a previous porcine study observed negative BOLD signals in motor cortex with high-frequency stimulation (130 Hz) of the ventral lateral thalamus and positive BOLD signals in the same cortical area with low-frequency stimulation (10 Hz). Moreover, the pattern of stimulation-induced activations in that study changed considerably as a function of stimulation frequency (Paek et al. 2015).

In addition to thalamic stimulation studies, the relationship between stimulation frequency and brain-wide activity has also been shown through electrical stimulation of parts of the basal ganglia in rodents (Lai et al. 2014). Globus pallidus stimulation with frequencies in the range of 40–160 Hz lead to increased hemodynamic signals in somato-motor cortex, unlike low (10 Hz) and very high (>190 Hz) stimulation frequencies. Optogenetic stimulation of the rodent hippocampus, on the other hand, revealed only relatively minor changes in signal strength as a function of stimulation frequency (6–60 Hz) (Weitz et al. 2015). Only the lowest frequency (6 Hz) resulted in weaker activations in connected sites. The lack of pronounced frequency-dependent effects may be due to the relatively small range of frequencies tested in the latter study, although we observed significant stimulation-induced fMRI effects between 10 and 20 Hz and between 50 and 100 Hz in the present study. In general, our results revealed a more complex picture for the effects of electrical stimulation when stimulating the VTA in monkeys at different frequencies: both the pattern of activations (Figs 5*a* and 6*a*), as well as the strength of these activations (Figs 5*b* and 6*b*) changed considerably as function of frequency. During the awake scans, middle frequencies (20–50 Hz) were most effective at inducing positive signal changes in distant sites. Remarkably, several areas showed opposite signal changes in going from 10 to 20 Hz and from 50 to 100 Hz (whereby negative and positive changes became positive and negative changes, respectively).

In humans, deep brain stimulation (DBS) has become one of the major clinical applications for altering brain activity to alleviate the symptoms of a number of brain disorders (Benabid et al. 2002). Although the relationship between stimulation frequency and therapeutic benefits is a complex topic that has not been extensively researched, there are clear examples of frequency-dependent clinical outcomes. For example, high-frequency stimulation (e.g., 130 Hz) of the subthalamic nucleus is exceedingly effective for tremor suppression in Parkinson’s patients, while low frequencies (<50 Hz) worsen such symptoms (Delong and Wichmann 2012). Speech, on the other hand, can be improved with low-frequency stimulation of the same nucleus (Abeyesekera et al. 2019). Such profoundly different results on behavior can be explained by frequency-dependent network effects, exactly as we observed in the present study. Indeed, the heterogeneous functional-network effects observed more broadly across animal studies corroborate the behavioral-outcome variability present in clinical studies. Moreover, frequency-dependent stimulation effects may vary across stimulation targets, which emphasizes the need to improve our understanding of frequency-dependent stimulation effects at a systems level.

In addition to frequency dependency, we found profound differences in stimulation effects between the anesthetized and awake states, unlike results described for stimulation of the dLGN (Logothetis et al. 2010) and parietal cortex (Premereur et al. 2015). These 2 monkey studies revealed no major state-dependent differences in stimulation-induced activity patterns, except that higher stimulation currents were required in the anesthetized preparations. Based on these results, we also increased the stimulation amplitude in the anesthetized animals, but found that the patterns of activation differed between states. While the lowest frequency yielded no effect or even deactivation in most areas when the monkeys were awake, this same frequency led to slightly increased activations in some ROIs under anesthesia. In contrast, higher frequencies increased and reduced activity in the awake and anesthetized state, respectively (compare Figure 3 with Figure 4, and Figure 5 with Figure 6). Our anesthetized data are the opposite of those observed in V1 after dorsal lateral geniculate nucleus (dLGN) stimulation (Logothetis et al. 2010). In that study, stimulation with the lowest frequencies (<48 Hz) deactivated V1, while higher frequencies (>48–200 Hz) led to positive BOLD changes in striate cortex. Moreover, stimulation of the globus pallidus and subthalamic nucleus has shown positive BOLD signals using higher frequencies (40–160 Hz) in somato-motor cortex (Lai et al. 2014). Note that we used ketamine which blocks NMDA receptors, while the dLGN stimulation study of Logothetis et al. (2010) relied on the opioid receptor agonist remifentanil, that does not lead to a general loss of consciousness, unlike ketamine. Therefore, the differences we observed may depend on the specific anesthetic used. Despite this, patterns of fMRI activation induced by cortical stimulation are similar between awake and ketamine anesthesia (Premereur et al. 2015). This indicates that the site of stimulation may affect whether an interaction with ketamine occurs. To confirm that the VTA-EM-induced activity patterns and changes in functional connectivity under ketamine are truly the result of differences in state, instead of being specific to ketamine, future experiments must be performed that compare alternative anesthetics (such as sevoflurane and propofol) with concurrent VTA-EM. Nonetheless, the differences in the functional connectivity profiles are in line with previously acquired functional connectivity profiles under ketamine anesthesia and sevoflurane (Uhrig et al. 2018). The general reduction in negative correlations under ketamine suggests that large-scale changes in correlated activity also influences the pattern of activity induced by VTA-EM.

Importantly, rodent studies during which the VTA is stimulated, while concurrently carrying out fMRI are typically performed under anesthesia (Decot et al. 2017; Lohani et al. 2017; Brocka et al. 2018), and we are not aware of other studies directly comparing VTA stimulation in awake animals, as we have done here. The discrepancy between awake and anesthetized preparations could be attributed to several factors. First of all, anesthesia can have a general effect on the pattern and amplitude of the recorded fMRI signals (Barttfeld et al. 2015; Tang and Ramani 2016). In addition, the VTA may be a particularly peculiar target, as it could play a central role in maintaining or switching mental states. Indeed, it has been shown that VTA stimulation can lead to reanimation from general anesthesia (Solt et al. 2014; Taylor et al. 2016). Therefore, the different activation patterns we observed between the anesthetized and the awake state might be the neural signature of altered network interactions required to switch between unconscious and conscious states. Yet, regardless of its causes, the interaction between the site of stimulation and type of anesthetic must be regarded as an important factor in determining the effects of frequency-dependent stimulation.

The profound differences between VTA stimulation and thalamic or cortical stimulation effects may be explained by the connectivity and functional roles of the targeted structures. The thalamus and early cortex are mainly drivers of activity in upstream areas. The ventral midbrain, on the other hand, is thought to modulate, rather than drive, postsynaptic activity. Also, the patterns of anatomical connectivity from selected stimulated sites in the dLGN and cortical areas are relatively small and confined, whereas that of the VTA is more diffuse, targeting virtually the entire neocortex with an emphasis on frontal areas (Berger et al. 1988; Bergson et al. 1995; Mark Williams and Goldman-Rakic 1998). Irrespective of the reason for these differences, it is clear that frequency and state-dependent EM effects cannot be generalized from area to area.

Intriguingly, we found substantially stronger stimulation effects in visual and premotor compared with prefrontal cortex, indicating that the strength of anatomical connectivity does not necessarily predict functional consequences of VTA microstimulation (Lohani et al. 2017). This is in contrast with earlier studies combining fMRI with either electrical or optogenetic stimulation targeting cortical (Ekstrom et al. 2008; Gerits et al. 2012) or thalamic sites (Logothetis et al. 2010), showing surprisingly strong correspondences between anatomical and effective connectivity. It should also be noted that, while VTA microstimulation does trigger dopamine release in primates (Schluter et al. 2014; Sander et al. 2019), all cell types surrounding the electrode will be affected. Therefore, our results cannot be directly attributed to DA signals. Recently, it has even been argued that non-DA neurons (e.g., glutamatergic or GABAergic) contribute more strongly to hemodynamic effects after stimulation than do DA neurons (Brocka et al. 2018). In addition, it needs to be recognized that dopamine binding on D_1–5_ receptors of microvessels and D_3_ receptors of astroglia can affect fMRI signals in a non-neural manner (Choi et al. 2006). In this respect, dopamine-specific stimulation through cell-type specific optogenetics (Stauffer et al. 2016) may be a more optimal method to study DA signaling from the monkey’s VTA. Nonetheless, cell-type specific optogenetic stimulation remains a difficult, low yield technique in primates (Galvan et al. 2017), which made VTA microstimulation an attractive alternative to examine the relationship between the frequency of VTA activity and widespread changes in neural activity.

Activations contralateral to the site of stimulation can be expected since the strength of anatomical connectivity of the VTA is surprisingly similar in the ipsi- and contralateral hemispheres (Geisler and Zahm 2005; Fox et al. 2016). Remarkably though, we observed substantial contralateral stimulation-induced activity, often even stronger than the homotopic ipsilateral activations. The observed contralateral dominance in the awake activation patterns is somewhat puzzling, since it deviated from our previously published findings in which we stimulated a different group of monkeys at a higher stimulation frequency of 200 Hz. In the latter study, we obtained strong ipsilateral activation in the animals as a result of VTA-EM (Arsenault et al. 2014). The asymmetry is, however, partially explained by the reduced tSNR in the ipsilateral hemisphere (Supplementary Material S3) due to the presence of the chronically-implanted electrodes and a recording well resulting in an asymmetrical placement of receiver coils. Importantly, the relationship between tSNR and sensitivity is inherent to time-series analysis, with reduced tSNR levels decreasing the capacity to detect significant signals changes (Parrish et al. 2000). Therefore, the lower tSNR reduces our capacity to detect ipsilateral VTA-EM activations. Note that relatively stronger modulations within the contralateral hemisphere were only observed during the awake sessions, where the tSNR asymmetry was greater.

Anatomically, the VTA is located close to the optic tract. A benefit of this proximity is that there is a behavioral indication (i.e., stimulation-induced eye movements (Ekstrom et al. 2008)) that signals when stimulation is activating collateral brain tissue surrounding the electrode. Moreover, stimulation parameters can be titrated such that eye movements no longer occur, indicating that current spread to adjacent structures is reduced. At the same time, eye movements must be carefully monitored, as small eye movements may elicit fMRI signal changes in visual, parietal, and frontal areas (Matsuda et al. 2004; Tse et al. 2010). In a pilot study, we did observe suspicious activity in these regions, which were probably induced by small eye movement induced by overly high currents (Supplementary Material S2). We therefore adapted our strategy for the present study, and used currents and frequencies that did not evoke measurable shifts in eye position.

Our examination of stimulation frequencies ranged between blocks of short (200 ms), high frequency (100 Hz) pulse trains to blocks of longer (2000 ms), low frequency (10 Hz) trains. The short trains were more comparable to phasic reward prediction error responses monitored during electrophysiology experiments in primates (Schultz et al. 1993), while the long trains represent a considerably more sustained modulation paradigm. Therefore, it is important to note that stimulation differed in both frequency, duration, and stimulated proportion of block (i.e., from more phasic at 100 Hz to more tonic at 10 Hz), and all of these features may contribute to the pattern of fMRI activity evoked by VTA-EM.

The precise way in which electrical stimulation frequency affects the local firing rates of individual or groups of cells remains an elusive matter. Nonetheless, optogenetic stimulation of rodent VTA shows that lower frequency stimulation elicits different firing rates in DA cells as compared with activity recorded at higher frequencies (Tsai et al. 2009; Lohani et al. 2019). Since the temporal signature of stimulation differentially influences the VTA locally, it is possible that this translates to a change in activity in its (in)direct projections. Hence, assuming that different simulation frequencies entrain different firing rates at the stimulated site, a speculative explanation for these findings may be that the VTA uses different frequency channels to communicate with different postsynaptic sites (Khamechian et al. 2019).

Interestingly, a recent study investigating the impact of VTA optogenetic stimulation on neural activity in the medial Prefrontal Cortex (mPFC) in rats lends some support to this idea. Phasic, fast-firing burst stimulation (100 Hz) of the VTA increased high gamma and high theta power for LFP recordings in the mPFC, whereas slow-firing burst stimulation (20 Hz) only increased low gamma power (Lohani et al. 2019). The underlying physiological nature triggering these differences in neural activity at projection sites remains unclear. Theoretical models highlight the complex temporal dynamics of DA release and its effect on projection sites, in particular when considering differential D1/D2 receptor distributions throughout the brain (Dreyer et al. 2010; Mandeville et al. 2013). More transient, phasic firing rates reduce the average D2 receptor occupancy and increase that of the D1 receptors compared with tonic firing. Moreover, optogenetic stimulation of the rodent VTA shows that more phasic-like stimulation (50 Hz) greatly increases DA levels in the NAc compared with tonic-like (1 Hz) stimulation as measured through voltammetry (Tsai et al. 2009). Therefore, it is conceivable that the amount of DA released in projection sites, differed over the range of frequencies at which we stimulated. Moreover, this indicates that the similar patterns of activity observed during the awake experiment at 10 and 100 Hz may result from different physiological processes. Overall, we do not assume that the physiological basis of our results are homogenous across frequencies and brain regions.

In summary, we observed substantial frequency and state-dependent stimulation effects on brain-wide activity consequent to targeting the VTA in monkeys. The frequency-dependent network effects may account for inconsistent clinical outcomes resulting from the different frequencies used in DBS therapies, and emphasize the importance of nonhuman primate models to investigate how the stimulation of various clinically relevant targets affects these targets within the brain.

## Funding

KU Leuven (C14/17/109); Fonds Wetenschappelijk Onderzoek-Vlaanderen (FWO-Flanders) (G0D5817N, G0B8617N, G090714N, G088813N and Odysseus G0007.12); and the European Union’s Horizon 2020 Framework Programme for Research and Innovation under Grant Agreement No 785907 (Human Brain Project SGA2). J.T.A. is a Postdoc fellow of the FWO.

## Notes

The authors thank C. Fransen, A. Coeman, P. Kayenbergh, I. Puttemans, C. Ulens, A. Hermans, G. Meulemans, M. Depaep, W. Depuydt, and S. Verstraeten for technical and administrative support and S. Raiguel for his comments on the manuscript. *Conflict of Interest*: The authors declare no Conflict of Interest.

## Supplementary Material

SupplementaryMaterial_bhaa007
