# Supplementary material for: Frequency- and State-Dependent Network Effects of Electrical Stimulation Targeting the Ventral Tegmental Area in Macaques
Source: Cereb Cortex. 2020 Apr 9;30(8):4281–96. doi: 10.1093/cercor/bhaa007 (PMC7325806; doi:10.1093/cercor/bhaa007)
Supplement: SupplementaryMaterial_bhaa007 [file supplementarymaterial_bhaa007.docx]

**Supplementary Material**

**Frequency and state-dependent network effects of electrical stimulation targeting the ventral tegmental area in macaques**

Sjoerd R Murris, John T Arsenault, Wim Vanduffel

|  | % Fixation during each of the experimental conditions | | | | | Kruskal-Wallis |
| --- | --- | --- | --- | --- | --- | --- |
|  | **No Stimulation** | **10 Hz** | **20 Hz** | **50 Hz** | **100 Hz** | **p-value** |
| Monkey T (65 runs) | 94.5 | 97.3 | 94.9 | 94.7 | 95.6 | p = 0.38 |
| Monkey D  (93 runs) | 97.3 | 98.0 | 97.2 | 97.9 | 97.7 | p = 0.86 |

**Supplement 1:** Statistical analysis of fixation performance over runs. The table reports percentage fixation performance within the online fixation window (Monkey T, 2.4 ± 0.6 by 5.1 ±1.1 visual degrees; Monkey D, 3.0 ± 0.3 by 2.9 ±0.4 visual degrees) during each epoch. The reported p-values are obtained using a non-parametric Kruskal-Wallis test across all conditions.


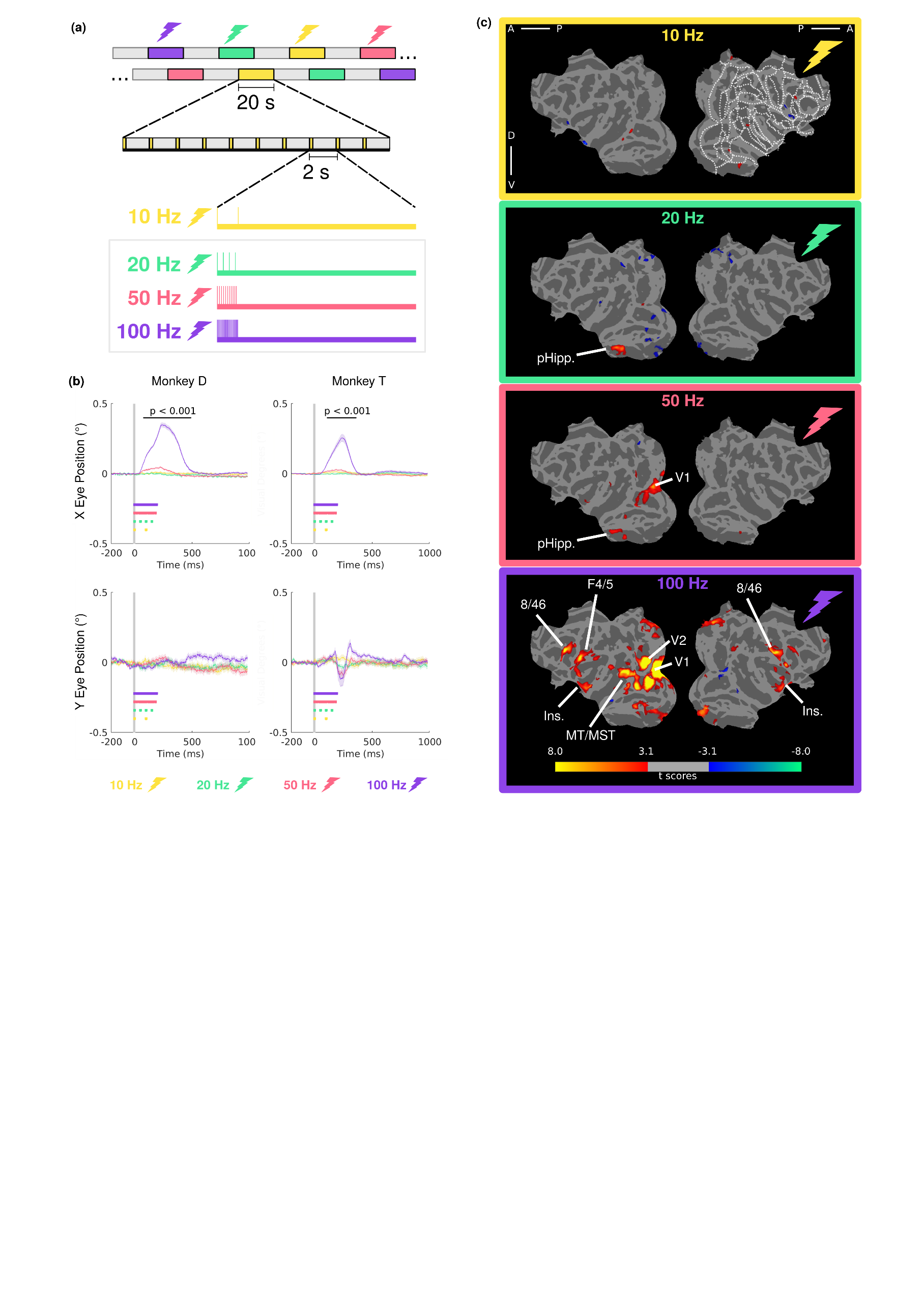


**Supplement 2:** Pilot experimental data in which stimulation-induced eye movements might partially explain the activity patterns. **(a)** Overview of the fMRI paradigm with 20 second stimulation blocks followed by 20 seconds rest (baseline). Each separate stimulation block consisted of 10 stimulation intervals with 200 ms stimulation at one of four frequencies (10, 20, 50 or 100 Hz). The order of the stimulation blocks was pseudo-randomized. **(b)** Eye-tracking data for both monkeys (Monkey T: 62 runs; Monkey D: 152 runs) along the horizontal (upper windows) and vertical (lower windows) dimensions. The four different stimulation frequencies are color-coded as indicated in the legend below panel b. Mean eye-positions across runs and SEM are plotted. Timepoints in which there is a significant difference between frequency conditions are indicated by the black horizontal line above the plots (ANOVA, p<0.001 with a Bonferonni correction for multiple comparisons). Notice clear VTA-EM induced eye movements for the 100 Hz condition and a trend for 50 Hz time-locked to the stimulation interval. **(c)** Activity maps for each of the four stimulation frequency conditions in a group analysis combining data from both monkeys (Monkey T: 4 sessions, 62 runs; Monkey D: 5 sessions, 152 runs). In the higher frequency conditions (50/100 Hz), activation of early visual cortex is probably related to eye movements.

|  | tSNR levels between hemispheres | | | | |  |
| --- | --- | --- | --- | --- | --- | --- |
| *Awake* | **M_left_** | **SD_left_** | **M_right_** | **SD_right_** | **p-value*** | **Δ M_left-_ M_right_** |
| Monkey D (60 runs) | 32.71 | 3.27 | 25.36 | 1.51 | 1.63 * 10^-11^ | 7.35 |
| Monkey T  (60 runs) | 25.54 | 2.32 | 21.99 | 1.94 | 1.63 * 10^-11^ | 3.54 |
|  |  |  |  |  |  |  |
| *Anesthetized* | **M_left_** | **SD_left_** | **M_right_** | **SD_right_** | **p-value*** | **Δ M_left-_ M_right_** |
| Monkey D  (30 runs) | 26.91 | 1.77 | 26.18 | 2.30 | 0.0082 | 0.73 |
| Monkey T  (30 runs) | 27.12 | 2.39 | 25.82 | 3.04 | 1.73 * 10^-6^ | 1.30 |

**Supplement 3**: To quantify potential inter-hemispheric differences in temporal signal to noise (tSNR), we compared the average tSNR signal averaged over all voxels from either the left or right hemisphere. Means (M) and standard deviations (SD) indicate tSNR across runs. The mean temporal SNR is always higher in the left hemisphere; in both monkeys, awake and anesthetized. The difference between hemispheres is statistically significant as indicated by p-values in the sixth column; *two-sided Wilcoxon Signed Rank Test (p<0.05). The right most column contains the difference in mean tSNR signal between hemispheres. When comparing states; the inter-hemispheric difference is more pronounced in the awake compared to the anesthetized monkeys. This difference is significantly stronger both in Monkey D (p = 1.37 * 10^-14^) and Monkey T (p =5.16 * 10^-14^) (p<0.05); two-sided Wilcoxon Rank Sum Test.


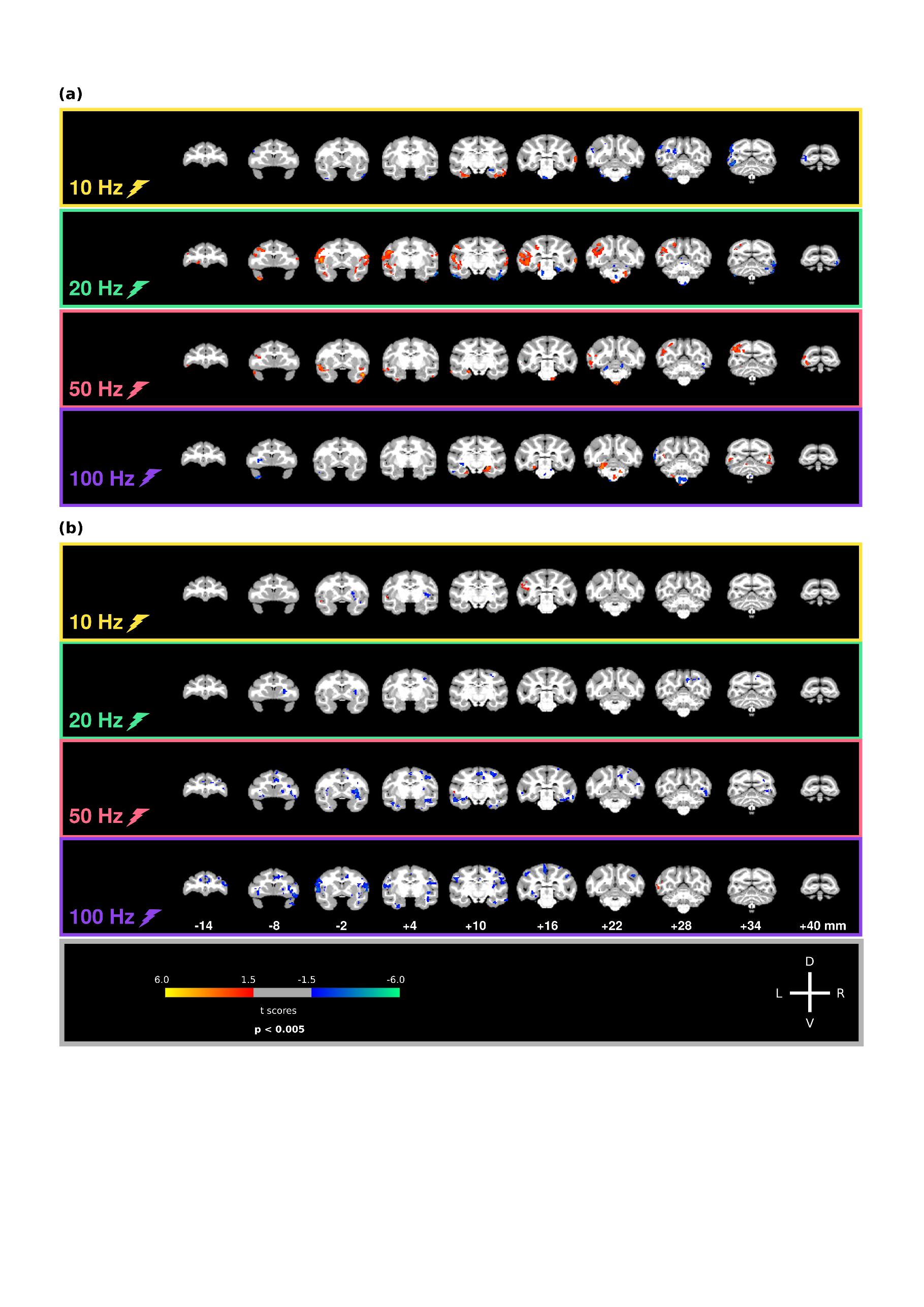


**Supplement 4:** **Conjunction analysis over monkeys of VTA-EM driven fMRI activity. (a)** Conjunction analysis of fMRI runs from awake sessions in monkey D (n = 60 runs) and monkey T (n = 60 runs) for each of the stimulation frequencies. **(b)** The same conjunction analysis applied to anaesthetized sessions for monkey D (n = 30 runs) and monkey T (n = 30 runs). The statistical significance of the conjunction analyses over both monkeys is indicated in the lower inset, together with the anatomical coordinates. The anterior-posterior position of the cerebral slices is relative to the anterior commissure (the zero position in D99 space).


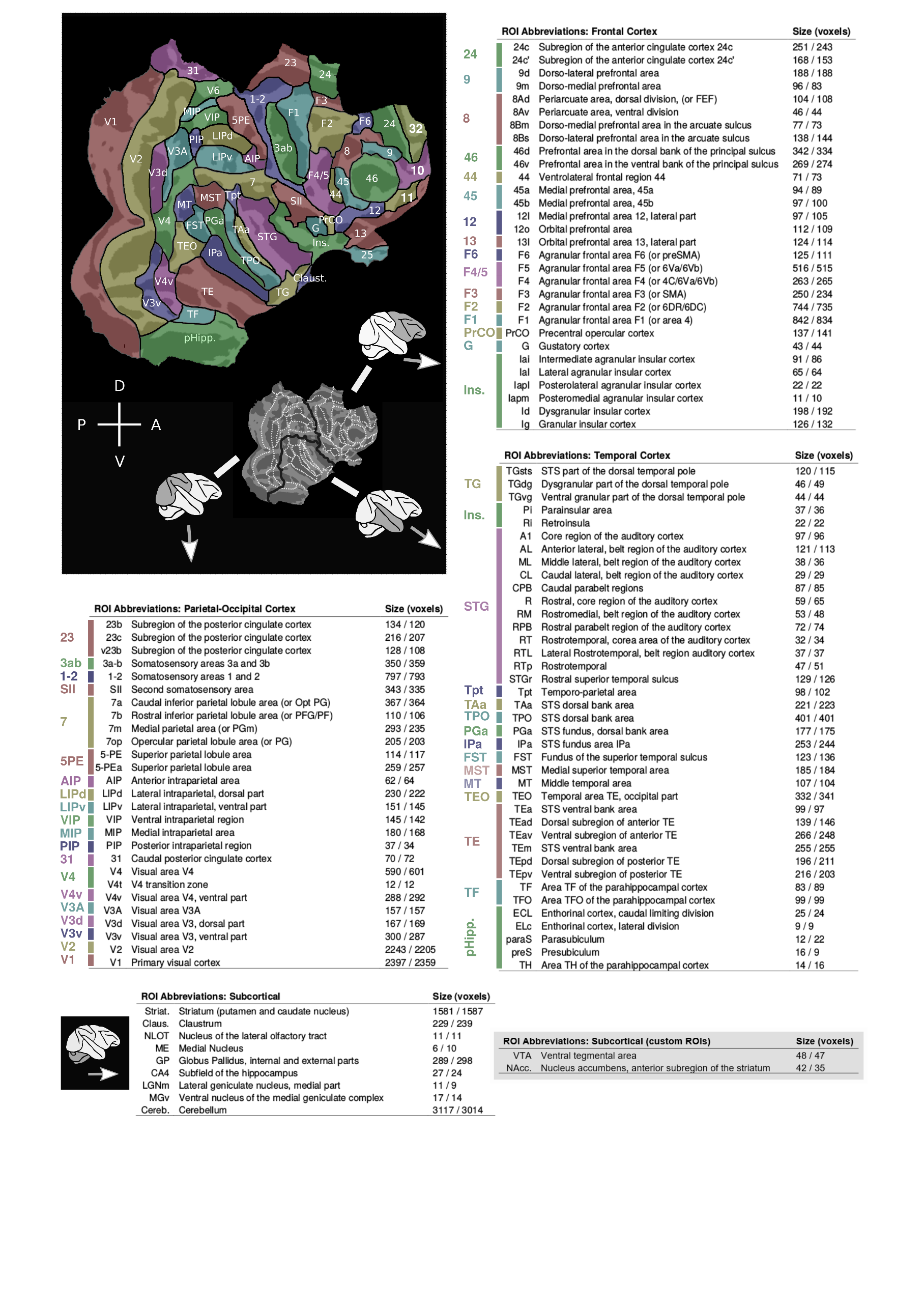


**Supplement 5:** Overview of ROIs in D99 template space, selected for our analyses. The enlarged flatmap of the right hemisphere (located in top left of figure) displays the location of all cortical anatomical ROIs. The smaller flatmap and associated cortical surface representations (lower right) display the translation of larger cortical subdivisions (e.g. temporal cortex) from 3D surfaces to 2D flatmaps. Each cortical subdivision has a table containing the abbreviations, description and the size of the ROIs (left/right hemisphere) it contains. The subcortical subdivision ROIs are presented separately at the bottom of the figure, including two custom made ROIs that are displayed in the grey box in the bottom right corner.
